# Esophageal perforation in South of Sweden: Results of surgical treatment in 125 consecutive patients

**DOI:** 10.1186/1471-2482-10-31

**Published:** 2010-10-28

**Authors:** Michael Hermansson, Jan Johansson, Tomas Gudbjartsson, Göran Hambreus, Per Jönsson, Ramon Lillo-Gil, Ulrika Smedh, Thomas Zilling

**Affiliations:** 1 Department of Surgery, Sahlgrenska University Hospital, Gothenburg, Sweden; 2 Department of General Surgery and Department of Thoracic Surgery, Lund University Hospital, Sweden; 3 Department of Cardiothoracic Surgery, Landspitali University Hospital, University of Iceland, Reykjavik, Iceland; 4 Department of Surgery, Varberg Hospital, Varberg, Sweden

## Abstract

**Background:**

For many years there has been a debate as to which is the method of choice in treating patients with esophageal perforation. The literature consists mainly of small case series. Strategies for aiding patients struck with this disease is changing as new and less traumatic treatment options are developing. We studied a relatively large consecutive material of esophageal perforations in an effort to evaluate prognostic factors, diagnostic efforts and treatment strategy in these patients.

**Methods:**

125 consecutive patients treated at the University Hospital of Lund from 1970 to 2006 were studied retrospectively. Prognostic factors were evaluated using the Cox proportional hazards model.

**Results:**

Pre-operative ASA score was the only factor that significantly influenced outcome. Neck incision for cervical perforation (n = 8) and treatment with a covered stent with or without open drainage for a thoracic perforation (n = 6) had the lowest mortality. Esophageal resection (n = 8) had the highest mortality. A CAT scan or an oesophageal X-ray with oral contrast were the most efficient diagnostic tools. The preferred treatment strategy changed over the course of the study period, from a more aggressive surgical approach towards using covered stents to seal the perforation.

**Conclusion:**

Pre-operative ASA score was the only factor that significantly influenced outcome in this study. Treatment strategies are changing as less traumatic options have become available. Sealing an esophageal perforation with a covered stent, in combination with open or closed drainage when necessary, is a promising treatment strategy.

## Background

A perforation of the oesophagus implies a serious therapeutic problem. If a mediastinitis develops the situation can become life threatening in a few hours. Strategies for aiding patients struck with this disease are changing as new and less traumatic treatment options are developing. The introduction of covered metallic esophageal stents (SEMS) has offered a less traumatic alternative. In this situation, when new methods are evaluated, it is important to have knowledge about how these patients have been treated in the past.

Treatment of esophageal perforations remains controversial and no consensus has been reached on the best treatment option. This is a reflection of the fact that this condition is difficult to study with a high degree of scientific power. The incidence of esophageal perforation is low and limited clinical materials are still reported. In 1997 when Brauer and co-workers published a review based on all publications on post-emetic spontaneous rupture of the oesophagus, more than 80% consisted of materials with fewer than ten cases [1].

At the University Hospital of Lund patients with esophageal perforation requiring surgery have been treated either at the department of general surgery or of thoracic surgery. This study presents the collected experience from both departments over a 36-year period. Even though treatment was influenced by aetiology and site of perforation, the extent of surgical treatment has varied over time. The aim of this study was to study how treatment strategies have changed over time at our hospital, to compare outcome in response to the treatment of choice, and to evaluate diagnostic efforts. Further, we wanted to try to identify prognostic factors that might have influenced outcome.

## Methods

During the period September 1970 to September 2006, 128 patients were treated at the University Hospital of Lund with a diagnosis of esophageal perforation. From 1970 to 1987 the patients were collected prospectively (n = 71). From 1988 to 2006 the patients were identified by searching both the local hospital register as well as the national hospital discharge register (National Board of Health and Welfare) (n = 57). Three records in the prospectively collected group could not be found and these patients were excluded. We studied the records of the remaining 125 patients and the following variables were recorded: age, sex, length of hospital stay, co-existing diseases, time from start of symptoms to treatment, site and cause of perforation, diagnostic modality, method of operation, complications and mortality. The American Society of Anaesthesiologists (ASA) score (1, healthy patients, no medical problems, 2 mild systematic disease, 3 severe systematic disease, but not incapacitating, 4 severe systematic disease that is a constant threat to life, 5 moribund, not expected to live 24 hours irrespective of operation) estimated by the anaesthesiologist was also recorded.

For esophageal stenting a covered Ultraflex^® ^stent (Boston Scientific) was used.

### Statistical methods

Data was expressed as median values, with minimum and maximum as range. The Chi-square test or Fisher's exact test was used to compare categorical data. Differences between two continuously distributed groups of patients were analyzed with the Mann-Whitney U-test or for more than two groups with the Kruskall-Wallis test. Survival rates were graphically depicted by Kaplan-Meier plots, and comparisons were made using a Cox proportional hazard model. We initially evaluated the following factors for potential impact on survival: age (continuous and categorical), decade of surgery (categorical), surgical procedure (categorical), pharyngostoma or not (categorical), site of perforation (cervical, thoracic upper, middle, lower as categorical), type of perforation (spontaneous or iatrogenic as categorical), thoracotomy or not (categorical), suturing of the perforation or not (categorical), time elapsed between perforation and surgery (continuous), co-morbidity (categorical), sex (categorical), ASA-score (categorical). In order to find the best model for the determination of survival a stepwise backward Wald procedure was used to eliminate non-significant impact factors. All reported p-values were two-sided, and p-values below 0.05 were considered to indicate statistical significance. Statistical analyses were performed with SPSS version 12 (SPSS, Chicago, Illinois).

The ethical committee of Lund University hospital approved the study.

## Results

**Figure 1:**
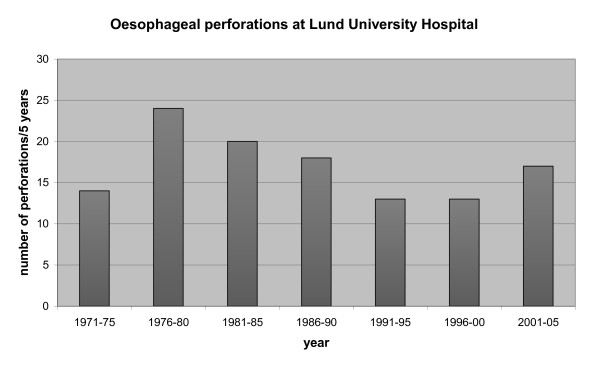
**Number of esophageal perforations at Lund University Hospital per five years**.

The median number of patients treated for esophageal perforation, requiring any form of surgical intervention, annually was 3 (0-8). When comparing consecutive five-year periods a maximum of 4.8 patients was treated yearly; this occurred during the period 1976 to 1980 (Figure1).

Basic data regarding the 125 patients are presented in Table 1. The male to female ratio was 62:38. The difference in sex ratio was significant comparing iatrogenic- and spontaneous perforations (p = 0.002) and regarding site of perforation i.e. cervical-compared to thoracic perforations (p = 0.009).

**Table 1:** Data on patients treated for an esophageal perforation from 1970-2006 at Lund University hospital

	Total n = 125	Cervical n = 15	Thoracic n = 110	Iatrogenic n = 70	Spontaneous n = 49
Sex					
male	77 (62%)	6 (40%)	71 (64%)^2^	36 (51%)	39 (80%)^1^
female	48 (38%)	9 (60%)	39 (35%)	34 (49%)	10 (20%)
Age (yrs)					
median (min-max)	65 (4-92)	67 yrs	64 yrs	65 yrs	61 yrs
ASA classification Pre-operatively					
mean	2,7	2,5	2,8	2,6	3,0
Mortality					
hospital	24 (19%)	1 (7%)	23 (21%)	14 (20%)	10 (20%)
<90 days post op	23 (18%)	1 (7%)	22 (20%)	14 (20%)	9 (18%)
Days in Hospital					
median (min-max)	21 (2-132)	16 (9-74)	22 (2-132)	16 (2-87)	35 (4-132)^3^
Cervical perforations				11 (16%)	0
Thoracic perforations	x	x	x	59 (84%)	49 (100%)
**Concurrent diseases**:					
Significant co-morbidity	36 (29%)	3 (20%)	33 (30%)	22 (31%)	13 (26%)
Benign esophageal disease	66 (53%)	5 (30%)	61 (55%)	48 (69%)	17 (35%)
Malignant esophageal disease	12 (10%)	0	12 (11%)	9 (13%)	3 (6%)

ASA score ranges from 1 (healthy) to 5 (moribund).

In three patients cause of perforation was foreign material and in three unknown.

^1 ^p = 0,002 ^2 ^p = 0,009 ^3 ^p = 0,001

The cause of perforation was iatrogenic in 70 cases and spontaneous in 49. Of the remaining cases the aetiology was foreign bodies (fish bones) in three while in three cases the cause was unknown (Table 2).

**Table 2:** Data on patients treated for an esophageal perforation from 1970-2006 at Lund University hospital

	1970-79 n = 36	1980-89 n = 37	1990-99 n = 27	2000-06 n = 25
Sex,				
male	17 (47%)	25 (68%)	21 (78%)	14 (56%)
female	19 (53%)	12 (32%)	6 (22%)	11 (44%)
Age				
median (min-max)	66 (15-90)	67 (4-83)	61 (44-88)	64 (24-92)
ASA classification pre-operatively (mean)	2,5	2,9	2,8	2,6
Mortality <90 days post op	10 (28%)	6 (16%)	5 (18%)	2 (8%)
Days in hospital				
median (min-max)	24 (5-87)	29 (5-132)	21 (3-67)	18 (2-101)
Iatrogenic perforations	26 (72%)	14 (38%)	14 (52%)	16 (64%)^1^
Spontaneous perforations	8 (22%)	22 (59%)	12 (44%)	7 (28%)
**Concurrent diseases**:				
Significant co-morbidity	7 (19%)	15 (40%)	6 (22%)	8 (32%)
Benign esophageal disease	20 (56%)	14 (38%)	17 (63%)	15 (60%)
Malignant esophageal disease	2 (6%)	3 (8%)	3 (11%)	4 (16%)

ASA score ranges from 1 (healthy) to 5 (moribund)

^1 ^p = 0,009

For iatrogenic perforations the hospital stay was significantly shorter than for spontaneous perforations, 16(2-87 days compared to 35(4-132) days (p = 0.009). We were unable to collect data concerning hospital stay and hospital mortality for one patient in each group.

The ASA patient status score was missing in 23 cases. Of these 23 patients one had a cervical perforation while 22 had thoracic perforations. With regard to aetiology, 10 perforations were iatrogenic, 11 were spontaneous, while two were caused by foreign material.

For the different time periods the missing ASA data were: 1970-79 seven, 1980-89 four, 1990-99 four and 2000-06 eight.

Table 2 shows the material divided into the time periods 1970 to 1979, 1980 to 1989, 1990 to 1999 and 2000 to 2006. There was a significant difference between the groups with regard to the distribution of iatrogenic and spontaneous perforations (p = 0.009).

A chest X-ray was performed on 44 patients (35%) and gave a suspicion of the diagnosis in 86%. Plain chest X-ray was considered "true positive" if there were findings that provided suggestive support for esophageal perforation, for example presence of mediastinal gas. The initial plain chest X-ray was complemented by contrast X-ray of the oesophagus or CAT-scan in most of the cases. Ninety-eight patients (78%) had a contrast X-ray of the oesophagus, which was true positive (93%). The corresponding figure for a CAT-scan with oral contrast was 95% (Table 3).

**Table 3:** Methods used to diagnose an esophageal perforation

Diagnostic tools	n:o of cases used n (%)	True positive n (%)	False negative n (%)
CAT-scan	22 (18)	21 (95)	1 (5)
Contrast plain film	98 (78)	91 (93)	7 (7)
Plain chest X-ray	44 (35)	38 (86)	6 (14)
Gastroscopy	5 (4)	4 (80)	1 (20)

Only eight percent of the patients had major surgery in the meaning of resection with primary reconstruction or exclusion. The majority of patients were operated upon with different kinds of drainage procedures as presented in Tables 4 and 5. Two patients are not presented in the tables. One died before a treatment decision had been made. One patient was operated trough an abdominal incision.

**Table 4:** Treatment strategies in esophageal perforations

*Number in figure 2*		1	2	3	4	5
***Thoracotomiced patients***	**All**	**+ Pharyngostoma+simple suture**	**+ Pharyngostoma**	**+ Simple suture**	**Only Drainage (+/- stent)**	**Esophageal resection or exclusion**

n (%)	99 (79)	13 (10)	20 (16)	34 (27)	24 (24)	8 (8)
Pre-operative ASA score						
mean	2,8	3,0	2,8	2,7	2,7	2,4
Post-operative complications	47 (47)	10 (77)	9 (45)	16 (47)	9 (38)	4 (50)
Re-operations	12 (12)	2 (15)	3(15)	3 (9)	3 (12)	1 (12)
Days in hospital						
median (min-max)	25 (3-132)	45 (16-67)	36 (5-132)	21 (5-70)	21 (3-102)	22 (4-87)
Hospital mortality	20 (20)	2 (15)	4 (20)	7 (21)	3 (12)	4 (50)
Mortality < 90 days post op	19 (19)	2 (15)	3 (15)	7 (21)	3 (12)	4 (50)

There were 3 patients in the group "only drainage" who received a covered stent. Among these patients there was one re-operation but no mortality.

ASA score ranges from 1 (healthy) to 5 (moribund)

**Table 5:** Treatment strategies in esophageal perforations

*Number i figure 2*		6	7	8
***Not thoracotomiced patients***	**All**	**Neck incision**	**Covered stent**	**Only conservative**

n (%)	26 (21) ^1^' ^2^	8 (6)	6 (5)	10 (8)
Pre-operative ASA Score				
mean	2,6	2,4	2,8	2,8
Complications	7 (23) ^2^	1 (12)	2 (33)	3 (30)
Late thoracotomy	4 (15) ^2^	0	2 (33)	1 (10)
Days in hospital median (min-max)	16 (2-126)	15 (9-32)	14 (12-17)	18 (5-126)
Hospital mortality	4 (15) ^1^	0	0	3 (30)
Mortality < 90 days post op	4 (15) ^1^	0	0	3(30)

^1 ^One patient died before treatment decision (diagnoses at autopsy).

^2 ^One patient was operated upon through an abdominal incision. This patient survived but had to be re-operated upon.

ASA score ranges from 1 (healthy) to 5 (moribund)

There were three patients in the group "only drainage" who received a covered stent. Among these patients there was one re-operation but no mortality.

**Figure 2:**
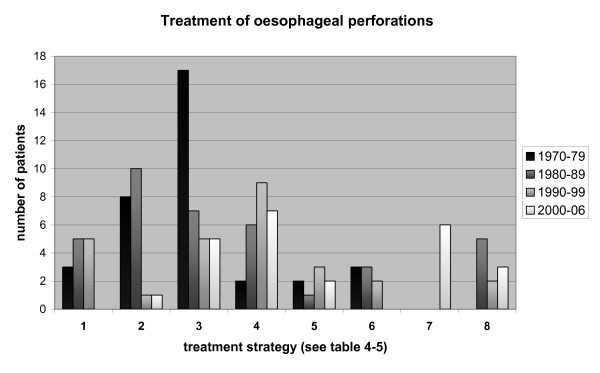
**Number of patients treated per decade according to the different strategies outlined in table 4-5**.

In figure 2 the numbers of patients treated according to each of the eight different treatment strategies (see Table 4 and 5) for each decade are presented.

In the group of patients who received intervention less than 24 hours after onset of symptoms 16 out of 71 died (22%). In the group of patients who were treated after 24 hours from onset of symptoms nine out of 48 died (19%).

When all potential impact factors for survival were compared and adjusted for in a multivariable Cox analysis, the only significant impact factor for survival was the ASA scores (p = 0.017).

## Discussion

### Treatment strategies

In this series we have identified eight different methods of treatment for esophageal perforations at the Lund University hospital from 1970 to 2006 (tables 4-5). The placement of a self-expandable covered metallic stent (SEMS) to cover the thoracic perforation, in combination with drainage if necessary, was associated with the lowest mortality in this study. It is, however, difficult to draw any firm conclusions regarding the effectiveness of this treatment modality since only eight patients received this treatment. The fact that mortality in eight patients after primary resection was 50% provides suggestive support for the notion that a minimally invasive procedure might be a better option after all. An interesting option in tumour perforations is to use a SEMS as a bridge to surgery and thereby avoid major surgery in the acute setting.

The construction of a pharyngostoma for diversion of saliva was frequently used at our hospital during the 70's, 80's and 90's as a safety precaution in combination with a suture of the perforation. A pharyngostoma, however, is very uncomfortable for the patient, and a second surgical procedure will be necessary to restore continuity. Since our results indicate that pharyngostoma after esophageal perforation is associated with longer hospital stay, and importantly, had no beneficial effects on mortality, we conclude that pharyngostomy should be used with restraint.

The treatment strategy for esophageal perforation in our hospital has changed markedly during the last decade (Figure 2). In the 70's, 80's and 90's an extensive surgical approach was used and approximately 25% of the patients received a pharyngostomy for diversion. Since 2000 only one out of 25 patients (4%) were operated upon in this manner and the most common operation today is sealing of the perforation endoscopically using a SEMS in combination with open or closed drainage when necessary. During the period 2000 to 2006, 52% of the patients were treated with open drainage without simple suture and/or a SEMS. As seen in table 2, both mortality and hospital stay are lower during this period than in previous periods, indicating that the change in treatment strategy may have improved outcome, although other factors, for example improved intensive care, may have contributed. One of the largest consecutive series published regarding SEMS treatment of esophageal perforations comes from Johnsson and co-workers in Gothenburg [2]. In this series all thoracic esophageal perforations from 1998 to 2004 (n = 22) was treated with a SEMS and favourable results are reported.

However, there are reports that cases of thoracic perforations without sepsis, can be treated conservatively with good results [3-5]. Primary repair with or without reinforcement is probably standard treatment for a perforation of the thoracic oesophagus at most centres. Some authors have advocated that primary repair should only be used in patients with early perforations and recommend resection or diversion when the perforation is older than 24 hours [6-8]. This has however been challenged by several groups that report good results with this technique even in patients who come to surgery late [9-13]. In cases of malignant disease in the oesophagus a resection should be performed [14-16]. In cases with severe damage to the oesophagus and severe contamination, an esophageal resection, diversion, exclusion or T-tube operation can be considered [6,7,17,18].

There are very little hard evidence published regarding the treatment of esophageal perforations, this is a disease that is difficult to study because of its low incidence and acute nature. We believe that this study indicates that the extensive surgical procedures, often including pharyngostoma, which was performed at our unit in the 70's, 80's and 90's should be used more moderately.

The acute surgical treatment of this condition includes two major aspects, sealing the perforation and drainage. This can be accomplished with conventional surgical intervention but often endoscopic methods, in combination with interventional radiology, are sufficient. It is important to understand the heterogeneity in this group of patients. In a patient with an iatrogenic perforation that is diagnosed immediately a SEMS in combination with a thoracic drainage tube is likely to be sufficient if the perforation is not too large. In spontaneous perforations of the distal oesophagus with severe contamination of the pleural cavity, surgery is often a better option in order to remove debris and because SEMS sometimes do not provide sufficient sealing in this area when its distal part dip into the stomach. In cervical perforations with no sign of mediastinal contamination drainage alone is the method of choice initially. Localization, size, degree of contamination, elapsed time since perforation and the patients general condition are all parameters that needs to be considered before a treatment decision is made.

### Diagnostic tools

One aim of the present study was to examine the effectiveness of different diagnostic tools. According to our data, reaching a diagnosis is rarely a problem if the suspicion of an esophageal perforation is raised. In our series, a CAT scan or an investigation with contrast X-ray of the oesophagus had the best sensitivity and specificity (Table 3). However, even a simple chest X-ray often contributed to the diagnosis. Considering the advances of the CAT-scan during recent years, this method in combination with contrast in the oesophagus should be the method of choice when an esophageal perforation is suspected. With contrast in the esophagus it is easier to detect and estimate the size of the defect. A large amount of contrast medium in the pleural cavity indicates a large perforation with heavy contamination. At our centre we frequently perform an endoscopy as a complement before the final treatment decision in order better evaluate the size and location of the perforation.

### Prognostic factors

Pre-operative ASA score was the only factor that significantly influenced outcome in the Cox proportional hazards model. Age had a major influence though its effect did not reach the level of statistical significance. To our surprise, cause of perforation and time interval from symptom to treatment did not significantly influence outcome in this study, even though others also has reported this lack of correlation [8,11,19]. Spontaneous perforations did however have a longer hospital stay compared to iatrogenic perforations. In a review by Brinster et al [14] results from nine recent case series with a total of 431 patients, were studied. They found a higher mortality among patients with a spontaneous perforation (36%) compared to an iatrogenic perforation (19%). They also found an influence of the time factor on mortality in 390 patients from 11 series. If treatment was delayed more than 24 hours, mortality in these series was 27% compared to 14% if treatment was initiated within 24 hours. Even higher mortality figures with delayed treatment was reported by Brauer et al from a large literature review of Boerhaaves syndrome [1]. This is consistent with results from studies regarding gastric or duodenal perforations [20]. Although our study did not confirm the importance of these prognostic factors it is likely that they have some influence on outcome, particularly the time to treatment factor. According to our data, and not surprisingly, the general condition of the patient at the time of diagnosis is probably the most important prognostic factor. This is a heterogeneous material with all sorts of esophageal perforations. In the case of a small, contained perforation that does not rapidly cause mediastinitis, the diagnosis might be considerably delayed without a septic condition developing. On the other hand, a patient who does develop sepsis because of mediastinal and pleural contamination will be in poor condition even if treatment is prompt. This is supported by the results obtained in a previous multivariate analysis from the Netherlands [19]. In that study neither cause of perforation nor time interval significantly influenced survival. However, they report a marked difference in mortality if a perforation was confined to the mediastinum or if it had perforated the pleura. It would have been interesting to stratify our patients, other than ASA classification, according to the seriousness of the perforation. This was however difficult due to the retrospective design of this study.

Not surprisingly, cervical perforations had a lower mortality compared to perforations in the thoracic cavity. This is consistent with earlier reports [19,21-24]. The reason for the more benign course in proximal perforations is that mediastinitis often does not occur; infected material spreads slowly from the neck, through the retro-esophageal space, to the mediastinum. These patients can often safely be approached conservatively.

### Limitations of the study

This is a retrospective study with a long observation time span. Because many parameters have changed during this period, for example the quality of intensive care, it is very difficult to compare outcome between the different time cohorts. Another limitation is the fact that the material is heterogeneous. We believe, however that the material is too small to enable further stratification.

## Conclusions

Pre-operative ASA score was the only factor that significantly influenced outcome in this study. Sealing an esophageal perforation with a covered stent, in combination with open or closed drainage depending on the patient's condition, is a promising treatment strategy that we believe can be the future method of choice for treatment of this condition.

## Competing interests

The authors declare that they have no competing interests.

## Authors' contributions

MH: planning, collection of data, evaluation of data, writing article

JJ: planning, evaluation of data, statistical evaluation

TG: planning, collection of data, evaluation of data, assisted in writing article

GH: planning, collection of data

PJ: planning, evaluation of data

RL-G: planning, collection of data

US: planning, evaluation of data, assisted in writing article

TZ: planning, evaluation of data, assisted in writing article

All authors have read and approved the final manuscript.

## Pre-publication history

The pre-publication history for this paper can be accessed here:

http://www.biomedcentral.com/1471-2482/10/31/prepub
